# Ecological Momentary Assessment of Bipolar Disorder Symptoms and Partner Affect: Longitudinal Pilot Study

**DOI:** 10.2196/30472

**Published:** 2021-09-02

**Authors:** Mor Yerushalmi, Andrew Sixsmith, Ariel Pollock Star, David B King, Norm O'Rourke

**Affiliations:** 1 Department of Psychology Ben-Gurion University of the Negev Be'er Sheva Israel; 2 Science and Technology for Aging Research (STAR) Institute Simon Fraser University Vancouver, BC Canada; 3 Department of Public Health Ben-Gurion University of the Negev Be'er Sheva Israel; 4 Department of Psychology University of British Columbia Vancouver, BC Canada; 5 Department of Public Health and Multidisciplinary Center for Research on Aging Ben-Gurion University of the Negev Be'er Sheva Israel

**Keywords:** bipolar disorder, couples, dyadic analyses, ecological momentary assessment, EMA, bipolar disorder, partner, relationships, mHealth, mobile apps, mental health, depression, BPD, mood

## Abstract

**Background:**

The World Health Organization ranks bipolar disorder (BD) as the 7th leading cause of disability. Although the effects on those with BD are well described, less is reported on the impact of BD on cohabiting partners or any interactions between the two; this requires in vivo data collection measured each day over several months.

**Objective:**

We set out to demonstrate the utility of ecological momentary assessment with BD couples measured using yoked smartphone apps. When randomly prompted over time, we assumed distinct patterns of association would emerge between BD symptoms (both depression and hypo/mania) and partner mood (positive and negative affect).

**Methods:**

For this pilot study, we recruited an international sample of young and older adults with BD and their cohabiting partners where available. Both participants and partners downloaded separate apps onto their respective smartphones. Within self-specified “windows of general availability,” participants with BD were randomly prompted to briefly report symptoms of depression and hypo/mania (ie, BDS_x_), positive and negative mood (ie, POMS-15; partners), and any important events of the day (both). The partner app was yoked to the participant app so that the former was prompted roughly 30 minutes after the participant with BD or the next morning if outside the partner’s specified availability.

**Results:**

Four couples provided 312 matched BD symptom and partner mood responses over an average of 123 days (range 65-221 days). Both were GPS- and time-stamped (mean 3:11 hrs between questionnaires, SD 4:51 hrs). Total depression had a small but significant association with positive (r=–.14; *P*=.02) and negative partner affect (r=.15; *P*=.01]. Yet total hypo/mania appeared to have no association with positive partner affect (r=–.01; *P*=.87); instead, negative partner affect was significantly correlated with total hypo/mania (r=.26; *P*=.01). However, when we look specifically at BD factors, we see that negative partner affect is associated only with affrontive symptoms of hypo/mania (r=.38; *P*=.01); elation or loss of insight appears unrelated to either positive (r=.10; *P*=.09) or negative partner affect (r=.02; *P*=.71). Yet affrontive symptoms of hypo/mania were significantly correlated with negative affect, but only when couples were together (r=.41; *P*=.01), not when apart (r=.22; *P*=.12). That is, these angry interpersonal symptoms of hypo/mania appear to be experienced most negatively by spouses when couples are together.

**Conclusions:**

These initial findings demonstrate the utility of in vivo ambulatory data collection in longitudinal mental health research. Preliminary analyses suggest different BD symptoms are associated with negative and positive partner mood. These negative effects appear greater for hypo/mania than depressive symptoms, but proximity to the person with BD is important.

## Introduction

### Background

One clinical feature of bipolar disorder (BD) is variable awareness of symptoms, their severity, and impact on others; this appears especially true when manic [1]. Fortunately, smartphones today enable active and passive measurement of mood and behavior for those with mental health conditions [2-4], including BD [5-7]. Both prompted data collection [8], and embedded sensors [9] enable smartphones to capture, synthesize, and share information from those with BD and their carers (eg, spouses) [10].

For the bipolar affective disorders and older adults (BADAS) study, we randomly prompted and measured BD symptoms in the moment [11]. For this pilot study, a subset of BADAS participants with cohabiting partners downloaded the carers app onto their smartphone. We set out to demonstrate the viability of dyadic ecological momentary assessment (EMA) and to compare BD symptoms (both depression and hypo/mania) and partner mood (positive and negative affect) over time.

For this pilot study, we had two specific aims. First, to demonstrate the utility of in vivo*,* ambulatory assessment with BD couples (ie, yoked smartphone apps). Essentially, would persons with BD and their cohabiting carers regularly provide subjective information when randomly prompted by their respective smartphones? Assuming that ambulatory data collection proves effective, are BD symptoms and partner mood correlated when measured each day over several months? And if so, in which directions (eg, depression correlated with negative partner affect)?

Data collection via smartphone app allowed us to determine who responded first each day (i.e., participant then his or her partner; or partner then participant), the interval between their respective responses, and whether they were together or apart (ie, shared vs distinct GPS coordinates).

### BD Symptom Measurement

With few exceptions, BD symptom scales rely on both self-report and memory (eg, recall over the past week or month) [12]. Yet research indicates that retrospective responses are affected by recall (eg, forgetting) and various response biases [13]. For instance, end-of-day retrospective reports capture just 26% to 37% variability in mood compared to in-the-moment responses obtained earlier that day [14]. Moreover, recall accuracy declines at times of increased life stress.

This has fostered observational and objective measurement, while euthymic and symptomatic [15,16] and where people with BD work and live [17,18]. Initial research suggests that the use of smartphones can foster self-insight and help forestall BD mood episodes when patients are medication adherent [19]. This is possible because smartphones are ubiquitous today and can measure, store, and transmit data in real-time, along with location and biometric data [20-22]. This allows us to identify person-specific factors associated with the onset and maintenance of BD mood episodes [23], including the ability to sustain supportive relationships, which are important to wellness with BD over time [24].

### BD Carer Well-Being

BD affects not only those diagnosed but also their family, friends, coworkers, and neighbors [25,26]. The negative impact of BD on carers includes mood episodes (depression and hypo/mania) [27], financial problems [28], and reduced social and functional well-being [29]. As a result, quality of life for BD carers can be severely impacted [30]. Compared to those caring for those with major depression, BD carers report greater burden and role strain [31].

According to Reinares et al [32], carer burden is greatest when those with BD are agitated, irritated, and depressed. Yet suicidal ideation causes carers greatest distress [33]. One challenge for BD carers is loss of control as BD mood episodes are generally unannounced, patients can present with depression, hypo/mania, or both [34], and recovery between episodes is often incomplete [35-37].

Though research examining the impact of BD on friends and family has grown in recent decades [38], all studies to date are based on retrospective questionnaire responses [31,39] or limited by very small sample sizes due, in part, to the low BD prevalence [40]. Social media recruitment for the BADAS study enabled the enrollment of an international sample of young and older adults with BD and their cohabiting spouses or partners when available.

## Methods

### Study App Development

The BADAS study app and data collection platform were developed, tested, and refined over 2 years, including iterative pilot testing in the field to ensure the app functioned as intended and data are reported as recorded (eg, GPS coordinates corroborated by self-reported location). Pilot testing occurred across multiple locations and time zones [8].

### BADAS Study Recruitment

We first recruited 50 adults with BD living in Canada, the United States, the United Kingdom, South Africa, and Australia. Participants were recruited using microtargeted social media advertising drawn from a global population of 6.2 million English-speaking, adult Facebook users with ‘bipolar disorder interests’ (eg, members of online BD support networks). As described elsewhere in more detail [41], machine-generated algorithms calculated by social media platforms are unique not so much for their sensitivity but specificity (ie, exclusion of those who do not have BD). Thus, persons recruited via Facebook do not represent the population, but we can be confident these are persons with BD because only persons with BD received the advertisements.

After clicking the ad, prospective participants were directed to a website describing the study; if eligible and interested, they were asked to provide their names and contact information. During screening interviews (telephone, Zoom, or Skype), prospective participants confirmed their BD diagnosis and provided emergency contact information (eg, psychiatrist). This was prudent, as bipolar disorder has the highest rate of suicide of all mental health conditions [42]. Ethics approval for this study was provided by Simon Fraser University, Burnaby, British Columbia, Canada.

### Partners of Persons with BD

Participants were also asked if they currently lived with a spouse or partner and to provide their partner’s email address. Only with the participant’s permission did we send email requests to their respective partners, inviting their participation. Both were assured that no information would be shared between them. Despite this, most BADAS participants requested that we not invite their spouses or partners to take part. Only 3 women and 1 man agreed and downloaded the partner app onto their smartphones (eg, App Store). No partners were lost to attrition.

We purposefully recruited partners without mental health diagnoses; one couple, in which both partners had BD, was excluded. This allowed us to examine associations between normal affect and BD symptoms (ie, pathology).

### Instruments

**Figure 1:**
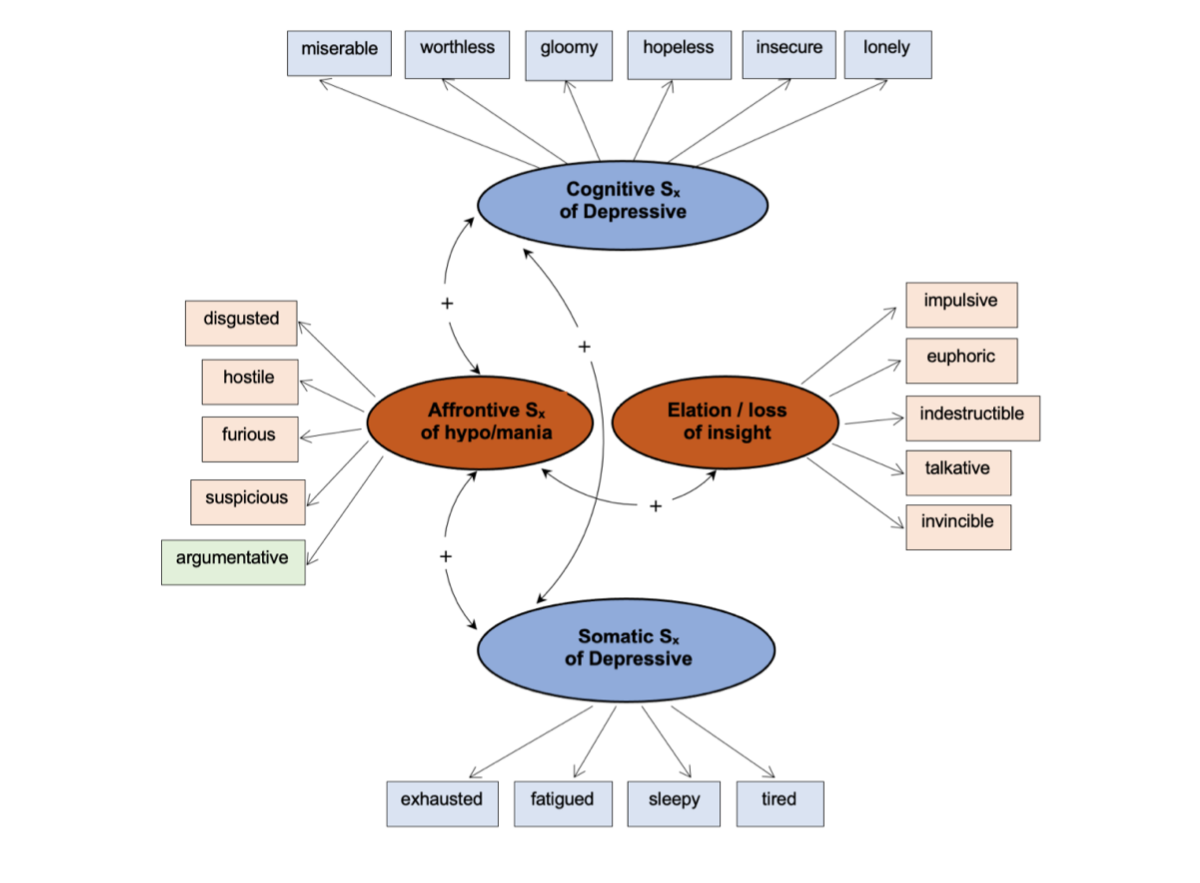
Four-factor model of bipolar disorder symptoms.

The bipolar disorder symptom scale (BDS_x_) [43] was developed for brief, ambulatory assessment of depression and hypo/mania. Respondents indicate the degree to which each of 20-mood adjectives corresponds to how they feel right now, at that moment. Research suggests a four-factor structure: two depression (cognitive and somatic) and two hypo/mania factors (elation or loss of insight and affrontive symptoms). The two depression and two hypo/mania factors are correlated, and affrontive symptoms of hypo/mania (eg, furious, disgusted, argumentative) are positively correlated with both depression factors suggesting pathways for mixed symptom presentation [44]. The construct validity of this four-factor model of symptomology was demonstrated across BD subtypes [45] and relative to quality of life with BD [46] (Figure 1).

The BDS_x_ was developed for ecological momentary sampling of BD symptoms via smartphone app [43,44] but has also been validated for use online [45,46] and as a printed-page screening measure with BD outpatients [47,48]. In this study, α=.88 for depressive symptoms and α=.71 for the hypo/mania subscale.

### Profiles of Mood States

Partner affect was assessed using the 15-item version of the profile of mood states (POMS-15), revised for daily diary research [49]. Participants are asked to rate each item on a Likert scale ranging from not at all (1) to a lot (3). POMS-15 items measure 12 negative and 3 positive emotions. This emphasis is based on research indicating that negative affect is (a) more reliably associated with individual functioning within context of acute stress and (b) more likely to be conveyed between partners and influence interpersonal processes than positive affect [48]. Internal consistency for negative POMS items measured over repeated points is high for paramedics and their spouses (.87<α<.90) [50]. In this study, α=.87 for negative affect and α=.74 for positive affect.

### Ecological Momentary Assessment

At recruitment, BADAS participants and partners specified “windows of general availability” in which they were randomly prompted to complete brief questionnaires on their respective smartphones. Participants were prompted twice daily to complete the BDS_x_ (AM and PM), describe sleep quality (AM), medication adherence (AM), and any important events of the day, the importance of the event, and its impact on mood and perceived control (PM).

Partners completed a single evening questionnaire that included the POMS-15. Positive and negative affect are inversely correlated but distinct aspects of mood associated with distinct brain regions [51]. Positive and negative affect are not endpoints along a single continuum. To our knowledge, this is the first study to examine positive and negative affect as distinct constructs relative to BD symptoms, and the first to measure the effects of affrontive symptoms of hypo/mania on cohabiting carers.

BADAS participants and partners were randomly prompted up to 3 times within 30-minute data collection windows. If they did not respond within the first 20 minutes, the app prompted them again. A third and final prompt was sent 5 minutes thereafter (if they did not respond to the second prompt). Participants could select a distinct or dedicated tone to distinguish study-related prompts from other smartphone sounds [8].

The partner app was yoked to their respective participant’s app to collect couple’s data within 30 minutes. When the participant responded later than their partner’s availability, they were prompted the next morning, before noon. Both the participant and their respective partner could submit voluntary questionnaires any time if they missed a prompted questionnaire or to report a particularly salient event in the moment. Both voluntary and prompted questionnaires were time- and GPS-stamped (ie, longitude and latitude), allowing us to determine if participants and partners were together or apart when their respective questionnaires were submitted.

### Participant Remuneration

BADAS participants were paid $1 CDN/day ($0.79 USD) if they complete both the AM and PM questionnaires when prompted. If they missed one AM or PM prompt (not both), they could later submit a voluntary questionnaire. Partners were also paid $1 CDN/day ($0.79 USD) on submission of a single PM questionnaire.

## Results

### Viability of Ecological Momentary Assessment with BD couples

For this pilot study, we identified 312 matched participant and partner app responses from 4 couples over an average of 123 consecutive days (mean 4 months and 3 weeks, range 65-221 days). This sample size is sufficient to detect medium to large effect sizes for correlation coefficients between BD symptomology and partner mood (where *d*=.80; α=.80) [52].

Although participants are few (N=4 couples), our ability to collect this volume of in vivo ambulatory data over an extended period supports our first research question (ie, N=312 matched responses). Specifically, data collection using yoked smartphone apps appears to be an effective method for long-term data collection from persons with severe mental illness and their carers (both prompted and ambient data).

### Correlational Analyses

Our ability to collect 312 matched responses from dyads demonstrates the efficacy of ambulatory data collection with BD couples over time (mean 123 days). BADAS participants submitted the BDS_x_ before their partners completed the POMS 45% of the time (139/312; mean 4:50 hrs, SD 5:34 hrs); but most days, partners provided responses before participants (173/312, 55%; mean 1:52 hrs, SD 3:42 hrs). This sequence was largely random as it began with the participant's PM prompt (ie, within specified PM availability). One or both responses might also have been reported voluntarily that evening, not as prompted questionnaires, which might also change the response order that day (ie, participant then partner vs. partner then participant).

This difference in completion intervals (1:52 hrs vs 4:50 hrs) reflects partners completing the questionnaire the next morning (ie, BDS_x_ submitted after partners were no longer available, following their instructions). This was not uncommon, and is consistent with the observation that those with BD are more likely to be night owls than early birds [53]. In contrast, when partners submitted the POMS first, BADAS participants also completed the BDS_x_ that evening.

We next examined correlations between total depression (cognitive and somatic symptoms), total hypo/mania (affrontive symptoms and elation or loss of insight), and partner mood (positive and negative affect). We found that depression had a small but significant association with positive (r=–.14; *P=*.02) and negative partner affect (r=.15; *P=*.01). Yet total hypo/mania appears to have no association with positive partner affect (r=–.01; *P=*.87); instead, negative partner affect was significantly correlated with total hypo/mania (r=.26; *P=*.01]. This coefficient is the largest in this table, suggesting that symptoms of hypo/mania affect partners more than depression. These preliminary findings suggest that symptoms of hypo/mania foster sadness (ie, negative affect), not reduce positive affect (Table 1).

**Table 1:** Correlation coefficients between bipolar disorder symptoms and positive and negative partner mood [N=312].^a^

	Positive affect	Negative affect	Total depression	Total hypo/mania
Positive affect, r (*P* value)	–^b^	*–.45 (.01)*	*–.14 (.02)*	–.01 (.87)
Negative affect, r (*P* value)	*–.45 (.01)*	–	*.15 (.01)*	*.26 (.01)*
Total depression, r (*P* value)	*–.14 (.02)*	*.15 (.01)*	–	*.29 (.01)*
Total hypo/mania, r (*P* value)	–.01 (.87)	*.26 (.01)*	*.29 (.01)*	–

^a^Statistically significant coefficients are in bold.

^b^Not applicable.

Consistent with existing research [47,48], depression and hypo/mania are positively correlated (r=.29; *P=*.01), suggesting that depression and hypo/mania are not inverse clinical states. Often participants reported both types of BD symptoms (eg, mixed features). By contrast, positive and negative partner affect are negatively correlated (r=–.45; *P=*.02).

### Partner Mood and BD Factors

Above, we noted that the largest coefficient between BADAS participants and partners in Table 1 is between total hypo/mania and negative partner affect (r=.26; *P=*.01). Yet when we look more closely at BD factors, we see that negative partner affect is associated only with affrontive symptoms of hypo/mania (r=.38; *P=*.01). Elation or loss of insight appears related to neither positive (r=.10; *P=*.09) nor negative partner affect (r=.02; *P=*.71; Table 2).

Similarly, we noted that cognitive symptoms of depression were significantly correlated with negative partner affect (r=.18; *P=*.01); however, negative affect appears unrelated to somatic symptoms (r=.03; *P=*.58). The inverse is seen with positive partner affect, which is inversely and significantly correlated with somatic symptoms of depression (r=–.20; *P=*.01) but not cognitive symptoms (r=–.05; *P=*.43).

**Table 2:** Correlation coefficients between positive and negative partner mood and bipolar disorder factors (N=312).^a^

	Positive affect, r (*P* value)	Negative affect, r (*P* value)
Cognitive S_x_ depression	–.05 (.43)	*.18 (.01)*
Somatic S_x_ depression	*–.20 (.01)*	.03 (.58)
Affrontive S_x_ of hypo/mania	–.10 (.07)	*.38 (.01)*
Elatio, loss of insight	.10 (.09)	.02 (.71)

^a^Statistically significant coefficients are in bold.

### Couples Together and Apart

As previously noted, symptom and mood questionnaires were time- and GPS-stamped when submitted, allowing us to determine when questionnaires were completed and if couples were together or apart (ie, same GPS coordinates). Cognitive symptoms were significantly associated with negative affect when together and apart, and somatic symptoms were inversely associated with positive affect. Elation or loss of insight was associated with neither positive nor negative mood. What might be described as classic or quintessential mania symptoms (eg, euphoria and impulsivity) appear unrelated to partner mood when couples are together or apart.

By contrast, affrontive symptoms of hypo/mania were significantly correlated with negative affect but only when couples were together (r=.41; *P=*.01), not when apart (r=.22; *P=*.12). This result supports the construct validity of this confrontation-related grouping of symptoms. Consistent with our operational definition, these angry interpersonal symptoms of hypo/mania are experienced most negatively by spouses when couples share the same GPS coordinates. The largest coefficient in these preliminary analyses is between affrontive symptoms and negative partner affect when together (r=.41; *P=*.01; Table 3).

**Table 3:** Bipolar disorder symptoms and partner mood (positive and negative affect) together and apart.^a^

	Positive affect, r (*P* value)		Negative affect, r (*P* value)	
	Together^b^	Apart	Together^b^	Apart
Cognitive S_x_ depression	.01 (.84)	–.22 (.13)	*.15 (.02)*	*.29 (.04)*
Somatic S_x_ depression	*–.18 (.01)*	*–.28 (.05)*	–.02 (.72)	.23 (.11)
Affrontive S_x_ of hypo/mania	–.05 (.39)	–.19 (.19)	*.41 (.01)*	.22 (.12)
Elation or loss of insight	.10 (.13)	.01 (.94)	.08 (.19)	–.07 (.60)

^a^Statistically significant coefficients are in bold.

^b^Participants and partners together when questionnaires submitted (ie, same GPS coordinates).

## Discussion

### Principal Findings

The objectives of this pilot study were to (1) demonstrate the viability of ambulatory data collection with BD couples and (2) identify associations between partner mood and BD symptomology over months of daily data collection. Both objectives were achieved. Moreover, preliminary analyses suggest distinct associations between depression and hypo/mania and positive and negative partner mood. GPS measurement enabled us to determine whether responses were submitted when couples were together or apart (ie, same longitude and latitude). Though recruitment and data collection did not occur as first intended, we largely met or exceeded the standards for ambulatory assessment recommended by Trull and Ebner-Priemer [54].

BADAS participant and partner apps were yoked so that responses from both would be received within 30 minutes, fearing that between-couple effects might dissipate after more than an hour. In other words, time intervals between reporting of BD symptoms and partner mood were longer than intended. However, this makes the number and size of coefficients within couples more noteworthy. For instance, BD symptom levels reported the night before remain correlated with partner mood the next morning, suggesting that the impact of BD symptoms on partners (or partner mood on participants with BD) is not limited to minutes but appears to persist for hours maybe days. Correlation coefficients between BD symptoms and partner mood are similar to coefficients reported between partners without mental illness [50,55,56].

We examined both positive and negative partner affect in relation to BD symptomology in real-time. This proved fortuitous as we found different associations between depression and hypo/mania and positive and negative partner affect. For instance, somatic symptoms of depression are inversely associated with positive affect, whereas cognitive symptoms of depression are significantly correlated with negative affect (not positive affect).

These results are largely consistent with previous research indicating that both depression and hypo/mania affect carer well-being [33]. Our findings have the advantage of measuring both participant symptoms and partner mood each day, close in time, and over several months. Ecological momentary sampling allowed us to collect responses in real-time, unaffected by recall biases, and in familiar settings (eg, home).

On average, we collected matched symptom-mood responses from couples each day over 3 months and 3 weeks (mean 123 days). By design, completion of app questionnaires required only 3-5 minutes. Brevity of measurement was integral to high participant retention and adherence. This high rate of participation may also be explained by participant remuneration for submission of app questionnaires. Notably, roughly 20% of BADAS participants opted to give their accumulated monies to a BD charity, suggesting both intrinsic and extrinsic motivation to participate in this study. Some participated to supplement their incomes, whereas others appeared to be motivated to contribute to BD research.

### Limitations and Future Research

The number, size, and pattern of coefficients we report warrant further study. More elaborate analyses of BD couple dynamics should be undertaken (eg, interactions and time-lagged effects) using contemporary analyses for daily diary analyses (eg, hierarchical linear modeling). Correlational analyses reported herein are preliminary. Most nonsignificant findings would be significant with larger samples; coefficients should be interpreted within ranges (eg, small correlation; .20<r<.35).

The primary limitation of this study is the sample size. We collected information from participants and partners over an extended period, but with only 4 couples; therefore, generalizability of findings is limited. This small sample size limits our ability to (ethically) report full descriptive information. Future study with more couples is needed to identify any gender or cross-national differences.

As noted above, the primary impediment to recruitment for this study was the reticence of BADAS participants to include their spouse or partner. Despite assurances that no information would be shared, the majority of participants asked that we not contact cohabiting spouses or partners. The reasons for this reluctance are not immediately apparent (ie, we did not directly ask). Future couples research should recruit partners first, then cohabiting persons with BD, to determine if this sequence proves more effective.

As recommended by Trull and Ebner-Priemer [54], instruments used in this study were developed and validated for ambulatory assessment. For instance, the BDS_x_ [43,44] was specifically developed to briefly measure both symptoms of depression and hypo/mania; and though we report good between-person reliability for both the BDS_x_ and the POMS-15, ideally, we should report both within-person and between-person reliability.

### Implications and Applications

Results of this study demonstrate the efficacy of EMA in dyadic mental health research. Though participants were few, we collected real-time information each day from couples over 4 months on average. Random data collection using smartphone apps is a viable methodology for longitudinal, dyadic research, including couples where one spouse lives with a chronic mental health condition. Due to the ubiquity of smartphones today, this yoked-app methodology can be applied to a range of mental health research applications. In addition, research is not limited to dyads as extended families and social networks should also be studied in vivo.

EMA data collection functioned effectively, allowing us to collect daily responses from couples when prompted. Yet allowing flexibility such as voluntary or unsolicited responses appears integral to data collection over extended periods. This, however, confounded our objective of collecting responses from both spouses within 30 minutes. Fortunately, results suggest that associations between partner mood and BD symptomology endure over extended periods (eg, the next morning). EMA research opportunities will continue to grow as mobile technology continues to advance.

EMA applications are not limited to research but also include self-care and care management. For example, push notifications (eg, SMS messages) can be generated in real-time, notifying those with BD and possibly their carers (eg, spouses) when responses suggest clinical symptomology. This can foster symptom awareness and help marshal the interpersonal resources needed to cope with and manage BD mood episodes more effectively.

## Abbreviations

BD: bipolar disorder
BADAS: bipolar affective disorders and older adults study
BDSx: bipolar disorder symptom scale
EMA: ecological momentary assessment
POMS-15: profiles of mood states
